# Causal Dynamics of Work Participation and Cognitive Health in Later Life: A Life Course Perspective

**DOI:** 10.1093/geroni/igaf122.189

**Published:** 2025-12-31

**Authors:** Xiaowen Han, Phyllis Moen

**Affiliations:** University of Minnesota, Minneapolis, Minnesota, United States; University of Minnesota, Minneapolis, Minnesota, United States

## Abstract

This study investigates how continued work participation shapes cognitive functioning in later adulthood, highlighting the timing, duration, and reciprocal nature of these causal processes within a life course framework. Drawing on data from the Health and Retirement Study (HRS), a nationally representative longitudinal survey of U.S. adults aged 50 and older, we employ advanced causal estimation methods—including marginal structural models, structural nested models, and a Bayesian model comparison algorithm—to disentangle the causal pathways between work engagement and cognitive outcomes while adjusting for time-varying confounders. We measure cognitive functioning with a 27-point scale capturing memory, working memory, and processing speed, and categorize work participation as not employed, part-time, full-time, or fully retired. Preliminary findings indicate that continued work participation exerts positive effects on cognitive functioning after accounting for dynamic confounders, with these protective effects becoming more pronounced at older ages, especially for individuals in non-managerial or non-professional jobs. We also observe reciprocity, as higher cognitive functioning increases the likelihood of remaining employed. By modeling time-varying confounders and comparing multiple model specifications, we capture both overall and subgroup-specific effects, illustrating how work participation in one’s 50s may influence cognitive status well into the 70s. Moving forward, we will employ a Bayesian model comparison algorithm to identify the best-fitting models across different measures of timing and duration of work participation, capturing these complex longitudinal causal dynamics. These results have important implications for public policy and organizational practices, particularly as aging societies face extended working lives and rising concerns about cognitive decline.

